# Partner notification for sexually transmitted infections in developing countries: a systematic review

**DOI:** 10.1186/1471-2458-10-19

**Published:** 2010-01-18

**Authors:** Nazmul Alam, Eric Chamot, Sten H Vermund, Kim Streatfield, Sibylle Kristensen

**Affiliations:** 1 ICDDR, B: Centre for Health and Population Research, Bangladesh, Dhaka, Bangladesh; 2 Department of Epidemiology, University of Alabama at Birmingham, Birmingham, AL, USA; 3 Vanderbilt University Institute for Global Health, Nashville, TN, USA

## Abstract

**Background:**

The feasibility and acceptability of partner notification (PN) for sexually transmitted infections (STIs) in developing countries was assessed through a comprehensive literature review, to help identify future intervention needs.

**Methods:**

The Medline, Embase, and Google Scholar databases were searched to identify studies published between January 1995 and December 2007 on STI PN in developing countries. A systematic review of the research extracted information on: (1) willingness of index patients to notify partners; (2) the proportion of partners notified or referred; (3) client-reported barriers in notifying partners; (4) infrastructure barriers in notifying partners; and (5) PN approaches that were evaluated in developing countries.

**Results:**

Out of 609 screened articles, 39 met our criteria. PN outcome varied widely and was implemented more often for spousal partners than for casual or commercial partners. Reported barriers included sociocultural factors such as stigma, fear of abuse for having an STI, and infrastructural factors related to the limited number of STD clinics, and trained providers and reliable diagnostic methods. Client-oriented counselling was found to be effective in improving partner referral outcomes.

**Conclusions:**

STD clinics can improve PN with client-oriented counselling, which should help clients to overcome perceived barriers. The authors speculate that well-designed PN interventions to evaluate the impact on STI prevalence and incidence along with cost-effectiveness components will motivate policy makers in developing countries to allocate more resources towards STI management.

## Background

Partner Notification (PN) for sexually transmitted infections (STIs) has been recommended as an important step in STI management to help interrupt transmission of infections, prevent potential re-infection, and prevent complications [1-4]. PN provides an opportunity to make index patients aware of risk-reduction strategies for avoiding STIs [5], enables earlier diagnosis for partners, motivate behavior change in clients and partners, and reduce the burden of disease in communities [6]. Three main approaches to PN have been suggested for STIs [7]: (1) provider-oriented notification methods that use third parties (usually health-care personnel serving as "contact tracers"); (2) patient-oriented notification methods that use index patients notify their partners, with or without the medication to actually treat the partner for the putative infection or infectious exposure [8], and (3) a mixed approach of contact-notification that engages the index patients to notify their partners, with an understanding that health-care personnel will notify those partners who do not present for treatment within a given time [7].

PN has come a long way since its inception in the 19^th ^century; however, it still has several issues in terms of efficacy, priority setting, adaptation of new approaches and cost effectiveness [4]. PN is not a standalone program in STI control and management, it almost always work as a complementary program with other routine activities including screening and treatment. There is wide variation in the policy and practice of PN approaches around the world [9]. No single PN approach has ever been found to be effective for all settings because of likely variations in STI rates and care structures. PN strategies that are effective and feasible in developed countries may not be applicable for developing countries [10]. Provider-oriented notification was found to be effective in reaching partners of people with STIs [11,12] but the evidence was mainly drawn from studies conducted in developed countries and also concurrent with the fact that provider- based notification is more expensive than patient-based approaches [13]. These high costs preclude provision of provider-oriented notification strategies for many developing countries and limit their application even where promulgated. Patient-oriented PN intervention strategies including patient-centered counseling [14] and patient-delivered medication were reported to increase partner referral in Africa [15]. However, implementation of STI PN programs remains limited in developing countries due to inadequate resources, poor infrastructure for diagnosis and management of STIs as well as social stigma [16-18]. Stigma and discrimination against people with STI undermine their ability to seek care, and their willingness to notify spouses or other partners of their STI infection status [19].

We sought to explore both the feasibility and the acceptability of PN in STI management in developing countries both from the demand side and the supply side perspectives. Our developing country focus acknowledges that PN approaches and outcomes are influenced by health system characteristics and resource limitations, legislation and policy, socio-cultural and socioeconomic factors, and stigma issues of importance to patients and their partners [10]. By developing country we referred to the low, lower middle and upper middle income countries based on World Bank's classification of economies based on gross national income (GNI) per capita [20]. We included only the PN studies on curable STIs (syphilis or gonorrhea or chlamydia or trichomoniasis), because HIV is not curable and there are special considerations for long-term support of behavioral change and access to medications. We targeted the following issues as a proxy to understand feasibility and acceptability of PN in developing countries: (1) Willingness of index patients to self-notify partners; (2) The proportion of partners notified or referred; (3) Client-reported barriers in notifying partners; (4) Staff or investigator-reported infrastructure barriers in notifying partners; and (5) PN approaches that were evaluated in developing countries.

## Methods

### Search Strategy

Three electronic bibliographic databases including Medline (via PubMed), Embase (via Scirus), and Google Scholar were systematically searched to identify relevant published articles on PN of STIs in developing countries. The reference lists of potentially relevant articles were examined for additional references and the "related" search key in PubMed was used from highly relevant articles to search for additional publications. We limited our automated searches to articles in English published between January 1995 and December 2007. Articles published before 1995 were not included as earlier findings may be less relevant to more recent PN intervention approaches and their outcomes, due to changes in STI diagnostics, medications, health care policies, and societal attitudes [21]. To identify a comprehensive set of possible search terms of PN for STIs, we consulted indexed terms in titles, key words and abstracts of published journal articles in this area. We searched eligible citations using (sexually transmitted infection or sexually transmitted disease) or (STD or STI) or (syphilis or gonorrhea or chlamydia or trichomoniasis), and (PN or partner referral or partner management or partner tracing or contact tracing or STI management) as key words. Searches were modified for Embase and Google Scholar databases to conform to their search structures. The first author exclusively performed searches under direct supervision of the last author (SK). Searches were updated through March 30, 2008.

### Screening of articles

In order to select a final set of articles for review, we examined the title and abstract of the articles and included articles if: (1) they were relevant to at least one of the five PN related issues highlighted above, (2) if the article was published from primary data, (3) if the data derived from a population in developing countries [20], and (4) if the articles dealt with PN of common curable STIs (gonorroea, chlamydia, syphilis, and trichomoniasis).

### Data abstraction

From each selected article, we abstracted information using a pre-specified 14 item data abstraction form. Abstracted data included authors name, year, study design, country, population covered, outcomes measures and major findings. Symmetrical to our review questions; quantitative outcome data were abstracted to determine the proportion of patients intending to self notify partners, the proportion of partners notified, and the proportion of partners reporting to the clinics for evaluation and treatment. We also synthesized qualitative textual information on barriers of notifying partners as reported by the clients. We further examined infrastructural barriers related to implementation of PN programs as reported by the program staff and/or the report authors. We considered the following criteria to assess the quality and relevance of the reviewed studies, whether a study: (1) had clear inclusion and exclusion criteria for study subjects; (2) followed a study design that allow generalizability of the studied subjects who were representative of a broader population at risk for STIs; and (3) used statistical tests point estimates with confidence intervals or p-values and adjusted for confounding and interaction when necessary. This quality assessment was done for our review purposes, not to include or exclude studies per se. "Higher-quality" studies were those having objectively measured PN outcome data, study subjects were randomly assigned and used proper statistical methods for analyzing data. "Moderate-quality" studies contained PN outcomes that were measured, but had study designs limiting the scope of generalizablity. "Lower-quality" studies had no clear indication of how study subject were recruited and outcomes were measured or reported only textual information on PN outcomes (Table 1 and 2).

**Table 1:** List of review articles by study type, population covered, and outcome measured (partner notification was investigated as a primary outcome in these studies).

References	Study design	Population and settings	PN outcome measured	Type of notification/counseling
[26]Clarke et al.2007 (Moderate)*	Survey with prospective follow-up	287 STI patients in peri-urban clinic in Peru	Past experience of PN, reasons for informing or not informing partner	Patient oriented notification Counseling by trained professional
[23]Desormeaux et al 1996 (Moderate)	Survey with prospective follow-up	23 pregnant women and 10 partners and 384 prenatal women in slum of Haiti	Partner referral proportions knowledge, attitudes of PN	Patient and provider oriented notification, No indication of counseling
[23]Díaz-Dlavarrieta et al 2007 (Low)	Cross sectional survey	209 women with syphilis and139 partners in maternity hospital in urban Bolivia	To understand association between PN and partner violence	Patient-led notification Trained social worker provided counseling
[18]Faxelid et al 1996 (Moderate)	Randomized trial	396 STI patients (94 women and 302 men) in urban clinic, in Zambia	If counseling is effective for improving partner referral	Patient-oriented notification counseling provided by female nurse and male clinical office
[25]Gichangi et al, 2000 (Moderate)	Cross sectional study	377 women with syphilis, in maternity clinic in Kenya	Proportion of partner notified and their determinants	Patient oriented notification. No indication of counseling
[62]Koumans et al, 1999 (Moderate)	Observational study	9552 STI patients in outpatient clinics in urban Central Africa Republic	Acceptability of patient-referral PN approach, and determine indicators	Patient oriented notification No indication of counseling
[24]Klisch et al.2007 (Moderate)	Qualitative study guided by psychological empowerment model	18 women diagnosed with antenatal syphilis in maternity hospital in urban Bolivia	Psychological empowerment factors corresponding to PN(intrapersonal, behavioral)	Patient-led notification A trained social worker provided counseling
[19]Liu et al, 2002 (Moderate)	Cross sectional study	406 men with STI in urban clinics in China	To understand factors related to partner referral	Patient oriented notification No indication of counseling
[14]Moyo et al. 2002 (Moderate)	Randomized trial and qualitative study	272 STI patients (135 men and 137 women) in public STD clinic in Zimbabwe	Effectiveness of single session counseling on partner referral	Patient oriented referral Health care worker provided counseling
[10]Mathews et al 2002(Moderate)	Pre and post intervention evaluation	335 STI patients, 185 in intervention group and 150 in health care clinic in South Africa	Self efficacy for partner referral and actual partner referral counseling	Patient oriented referral, Video based education
[16]Njeru et al 1995(Moderate)	Post intervention survey	254 STI patients (94 men and160 women) in primary level heath centre in Nairobi, Kenya.	Proportion of PN and referral	Patient-oriented notification Counseling provided by trained medical students
[17]Nuwaha et al. 2001 (Moderate)	Randomized trial	383 STI patients (187 women and 196 men) in urban STD clinic In Uganda	Efficacy of patient delivered partner medication approach	Patient-oriented referral Information, education and counseling by clinical officers
[22]Nuwaha et al. 2001 (Moderate)	Prospective cohort based on social cognitive model	426 STI patients (236 women and 190 men)in urban STI clinic in Uganda	Intention to refer partner and actual partner referral	Patient-oriented referral Patient-oriented referral Counseling provided by clinical officers
[15]Nuwaha et al, 2000 (Moderate)	Qualitative study, FGD and in-depth interviews	10 FGDs, and 40 in-depth district hospital in Uganda	To understand psychosocial factors associated with partner referral	Patient oriented notification No indication of counseling
[38]Sahasrabuddhe et al. 2002 (Moderate)	Survey with prospective follow-up	182 STI patients (157 male, 25 female) in district hospital in India	Intentions to notify partners and actual partner referral	Patient oriented notification did not mention who counseled
[34]Shumin et al. 2004 (Moderate)	Prospective cross sectional study	730 men with STIs in reference STI clinic in China	Proportion of patients willing to notify; proportion of partner referred	Patient oriented notification No indication of counseling
[37]Steen et al 1996(Moderate)	Cross sectional survey	427 patients with STI symptoms (325, women, 102 men) in primary care clinics, semi urban Rwanda.	Proportion of partner referral	Patient-oriented referral. Nurses provided prevention education
[47]Wakasiaka. et al, 2003(Moderate)	Survey of STI patients and providers	407 STI patients in urban primary care clinics in Kenya	Utilization of PN	Patient oriented referral, health care workers provided counseling
[27]Young et al 2007(Low)	Cross sectional survey within a randomized trial	626 women with STIs in urban clinics in South Africa	Acceptability and feasibility of two PN methods	Patient oriented notification and patient delivered notification No information on counseling

* Texts in parenthesis indicate quality ratings of the review studies as per our criteria mentioned in the methodology section

**Table 2:** List of review articles by study type, population covered, and outcome measured*

References	Study design	Population and settings	PN outcome measured	PN/counseling
[36]Boonstra et al, 2003 (Low)	Observational study	224 STI patients (89 men135 women) in primary care centers in Botswana	Proportion of patients given PN counseling	Patient oriented notification, Nurse or the Family welfare educator provided counseling
[52]Chilongozi et al, 1996 (Low)	Cross sectional survey	103 STI service providers and 150 STI patients in regional hospitals in Malawi.	Proportion of patients given partner referral advice	Patient oriented notification Medical officer, clinical officers provided counseling
[29]Faxelid et al 1997 (Moderate)	Pretest-posttest evaluation	400 STI patients, 200 each in intervention and control group in urban health centers in Zambia	Proportion of patients willing to notify or refer partners	Patient oriented notification, Trained counselor provided PN counseling
[40]Grosskurth et al, 2000 (Moderate)	Intervention evaluation studies	12895 cases with STI symptoms (5959 men, 6936 women) in rural health centers in Tanzania	Proportion of partner treated, Partner card given to all patients	Patient oriented notification, No indication of who provided health education
[30]Green et al, 1998 (Moderate)	Pretest-posttest evaluation	628 STI service providers in Jamaica	Proportion of STI given advice for PN	Patient oriented notification, No indication of counseling
[41]Hanson et al, 1997 (Low)	Observational study	59 STI patients (42 men, 17 women) in one urban and two rural clinics	Proportion of the providers offer PN counseling	Patient oriented notification Clinical rural clinics officer provided counseling
[31]Harrison et al 1998 (Moderate)	Descriptive study	Exit interview of 49 STI patients, 44 simulated clinic visits, 10 FGDs in rural area in South Africa	Availability and acceptability of PN initiatives	Patient oriented notification No indication of counseling
[54]Harrison et al 1997 (Low)	Qualitative study	15 in-depth interview STI clinic attendees in rural South Africa	Perceived beliefs and willingness of PN and referral.	Patient oriented notification No indication of counseling
[42]Jacob et al, 2004 (Moderate)	Cross sectional survey	405 men with STI symptoms and 129 drug stores or private clinics in Uganda	Proportion o and referred f partner notified	Patient oriented notification No indication of counseling
[39]Kamali et al, 2002 (Moderate)	Community based trial	8437 adult men and women in rural Uganda	Willingness to refer partner and partner treatment rates	Patient oriented notification No indication who provided counseling
[53]Lafort et al, 2003 (Moderate)	Observational study	215 client-contact observation and200 STI patients in family planning clinics in Cote d'Ivoire	Proportion of STI given advice Proportion partner notified	Patient oriented notification Midwives provided health education
[33]Malta et al, 2007 (Low)	Qualitative study	30 men and women with diagnosed STIs in 2 public clinics in Rio de Janeiro, Brazil	Proportion of STI patients given advice for partner referral	Patient oriented notification No indication of counseling
[64]Mathews et al, 1998 (Moderate)	Cross sectional study	331 STI patients (170 men, 161 women) in urban health centers in South Africa	Proportion of STI patients offered PN card	Patient oriented no Nurse and health educator notification provided counseling
[65]Mayaud et al, 1998 (Moderate)	Prospective follow-up study	12,534 men and women rural area in Tanzania	Proportion patient notified and referred	Patient oriented notification health education provided by trained health workers
[32]Manhart et al 2000 (Moderate)	Qualitative study	70 in-depth interviews of general population and STI providers in rural and urban Morocco	Awareness and acceptability of PN	Patient oriented notification No indication of counseling services
[35]Mertens et al, 1998 (Moderate)	Observational study	108 observed consultation for STI services in India	Proportion of patients were asked to refer partner or counseled	Patient oriented notification No indication of counseling
[43]Ndulo et al, 1995 (Moderate)	Descriptive study	179 STI patients (92 men, 87 women) in urban primary care centers in Zambia	Proportion of partner notified and treated	Patient oriented notification No indication of counseling
[44]Sano et al, 2004 (Moderate)	Descriptive study	female sex workers visited STD clinics in four provinces in Cambodia	Proportion of partner notified and treated	Patient oriented notification No indication of counseling
[45]Wang et al 2007 (Moderate)	Cross sectional study	1072 migrant population visited19 public STD clinics in three provinces in China	Proportion of partner notified by index men and women	Patient oriented notification No indication of counseling
[28]Wynendaele et al, 1995 (Moderate)	Pretest-posttest evaluation	299 STI patients (137 men, 162 women) in two districts hospitals in Malawi	Intention of partner referral before and after counseling	Patient oriented notification Trained counselor provided counseling

*partner notification was investigated as a secondary outcome in these studies

## Results

**Figure 1:**
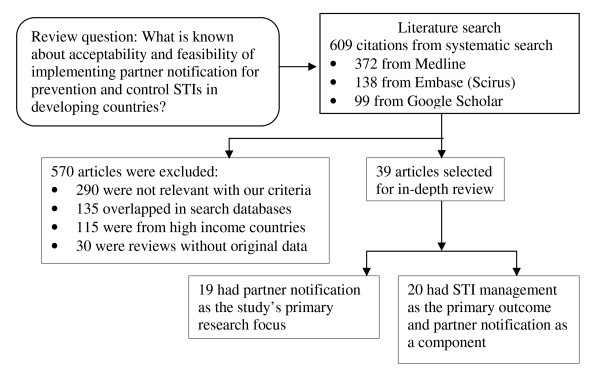
**Systematic review of published studies of perspectives and experiences with partner notification for sexually transmitted infections in developing countries**.

From 609 articles (474 unique: not overlapped across search data bases), 372 were from Medline, 138 from Embase (Scirus), and 99 from Google scholar data base. After the selection process (Figure 1), we identified 39 articles for this review, 28 from Africa, 6 from Asia and 5 from Latin American or Caribbean countries (Table 1 and 2). Studies had diverse study designs and populations. Only three studies were randomized trials [14,15,22]. Five studies collected data from women in antenatal clinics [23-26] or in home-based STI screening programs [27]. Four studies were pre-test post-test evaluation [10,27-30], five studies solely utilized qualitative design for data collection [15,24,31-33]. Other studies had either cross sectional design, observation or descriptive design and prospective follow-up design (Table 1 and 2). Two studies focused on men in STD clinics in China [19,34] and three studies collected data from STI service providers [30,35,36]. Other population covered in the studies included men and women from general population, pregnant women in antenatal clinics and medicine sellers. According to our quality assessment criteria, 32 articles were found to be of moderate quality, 7 were lower uality and none satisfy to be graded as higher quality.

### Willingness of index patients to self-notify partners

Seven studies described the willingness of index patients to self-notify their sexual partners (Table 3). Majority of the index patients expressed their willingness to self-notify their partners as indicated by their willingness based on a specific question in the survey or through accepting referral cards for their partners. The proportion raging from 58% to 93% [[14,23,37-40], except for one study reported that 77% of 406 men expressed their unwillingness to report their STI status to their spouse due to the associated stigma [19]. Motivation of index patients to self-notify their partners differed for men and women; many women felt certain that their husbands were the source of infection and therefore needed treatment. Pregnant women were particularly motivated to notify their spousal partner because they are concerned about infection risk to their fetus/baby [14]. Some women in Bolivia reported notifying their husbands to get information on the source of the STI and address infidelity in their marriage [24]. Men having STIs in Kenya reported notifying their wives to avoid infertility in themselves or in their wives, lack of condom use with wives as motivating factor for notification, fearing the likely spread of infection to their wives and their own reinfection [14].

**Table 3:** Partner notification outcomes by the research questions selected in this review.

**1. Willingness of an index STI patient to notify their partners**	
[23]Desormeaux et al, 1996	86% of the women intended to inform their partners of their STI status
[39]Kamali et al, 2002	59% of the patients received partner cards to notify partners
[19]Liu et al, 2002	only 23% of the married men with STIs expressed their willingness to inform their partners
[14]Moyo et al, 2002	93% motivated to reported their partners because women thought their husbands are source of infection and they need treatment or to save their upcoming child from infection, men were motivated to save their wives and themselves from infertility
[22]Nuwaha et al, 2001	women had higher positive intention (mean 1.3) to refer partner compared to men (mean 1.8)
[38]Sahasrabuddhe, 2002	78% intended to notify partners; among them, 80% women, 70% men.
[37]Steen et al, 1996	58% of the index patients accepted partner coupon for their partners
**2. Proportion of partners notified and/or referred to the clinics**	
[26]Clark et al, 2007	65% informed their primary partners, 10% informed their secondary partners
[23]Desormeaux et al, 1996	30% of the partners attended the clinics were referred by index patients
[60]Diaz-Olavarrieta et al, 2007	65% of the women reported to notify their partners
[25]Gichangi et al, 2000	94% partners were notified and 67% of the partner received treatment for syphilis
[40]Grosskurth et al, 2000	34% of the partner were notified and treated
[39]Kamali et al, 2002	25% of the partners of index patients received treatment
[24]Klisch et al, 2007	16 out of 18 participants (89%) notified their partners, 78% initiated treatment
[19]Liu et al, 2002	21% of the men reported to notify their partners.
[16]Njero et al 1995	68% notified their partners, 58% of the partners were reported to be treated
[34]Moyo et al, 2002	93% PN for spousal partner, 0% among causal partners
[17]Nuwaha et al, 2001	34% of the partners referred, 22% of the partners were referred by women and43% referred by men index patients
[37]Steen et al, 1996	45% of the partners got treated of those received referral cards
[38]Sahasrabuddhe, 2002	41% referred to the clinics; among then, 44% women and 40% men
[34]Shumin et al, 2004	23.3% partners were notified, 20.5% of the partners actually attended to the clinics and 13.3% received treatment
[45]Wang et al, 2007	46% of the women and 64% of the men informed their partners about their infections, PN was associated with higher rates of condom use and having had no commercial sex
**3. Barriers notified by the index patients**	
[26]Clarke et al, 2007	reasons for not disclosing STI status to primary partners included fear of rejection and embarrassment. for casual partners reasons were inability to locate partners and perception that informing a transient partner is not important
[40]Grosskurth et al, 2000	barrier included embarrassment, fear of violence and matrimonial conflict, the casual partners, and health workers did not explain the importance of partner treatment.
[25]Gichangi et al, 2000	women did not inform their partner because of fear of violence or being blamed for the illness.
[24]Klisch et al, 2007	reaction from male partner after notification included, silence and understanding, denying the possibility of having infection, blaming her for understanding, denying the possibility of having infection, blaming her for infection, becoming Aggressive with insults and shouting.
[14]Moyo et al, 2002	barriers for spousal partner included i. partner lived far away, ii. fear of loss of respect, embarrassment, iii. fear of divorce. for non spousal partners women indicated that they do not want to lose material support by annoying partners, for men, they would not have sex with them again.
[22]Nuwaha et al, 2001	barriers for women was their relationship will be known to the husbands and for men it was attitudinal beliefs that showing he is unfaithful, and ending of relationship or separation.
[15]Nuwaha et al, 2000	barriers for sexual partner referral were showing the partner that you are at risk of AIDS, creation of mistrust; showing unfaithfulness, refusal of sexual intercourse, ending of relationship, and separation or divorce.
**4. Structural barriers in implementing PN programs**	
[54]Harrison et al, 1998	only 3 clinics out of 10 used partner cards, 6 out of 10 clinics had a counselor
[16]Njero et al, 1995	lack of trained staff and inadequate infrastructure, mobile population, don't have any mail or telephone address, manually check records to verify partner referral outcomes.
[37]Steen et al, 1996	poor predictive values of STI diagnostics may lead to unnecessarily labeling of index patients and their partners with having STIs
[38]Sahasrabuddhe, 2002	limited resources and over burdened health system.
[34]Shumin et al, 2004	lack of staff especially availability of counselors across the STD clinics
[27]Young et al, 2007	lack of valid, user-friendly and cheap STI diagnostics
**5. PN approaches found successful in less developed countries**	
[18]Faxelid et al, 1996	1.8 partners per man were treated in counseling group compared to 1.2 in the control group (p < 0.001) but they did no counseling among women. find any significant effect of
[10]Methews et al, 2002	27% partners returned during video based intervention phase compared to 20% during control phase
[34]Moyo et al, 2002	92% partners were notified in counseling group compared to 67% in standard group, 93% referral for spousal partners compared to 0% for causal partner in case of male patients
[17]Nuwaha et al, 2001	74% partners were treated in patient delivered medication method and 34% treated in the patient based referral method
	for 85% of the partners, women accepted patient delivered medication and for 13% of the partners they accepted partner cards

### Proportion of partners notified or referred

Most studies report on PN outcomes as the proportion of partners notified and/or the proportion of partners reporting back to the study clinics. Nine studies reported the proportion of partners actually received treatment [16,27,33,39,41-45]. The median proportion of partners notified in the selected studies who reported quantitative estimates was 54% (range, 0% to 94%) depending on the type of partner and the means of verification (Table 3). These extremes were found in one study, where 0% notification for casual partners, but 94% for spousal partners [25]. The proportion of partners actually referred to the clinics was, 20% for female partners of male STI patients in China [34], 30% for male partners of women diagnosed with STIs in prenatal screening in Haiti [23] and 34% of the partners received treatment in patient oriented notification method in Zambia [22]. There was little correlation between the willingness of patients to notify their partners and their reported success; in one study 86% of patients were willing to notify, but only 30% of partners were reported as notified [23]. In another 58% of patients were willing to notify and 45% of partners were reported as notified [38].

### Client reported barriers in notifying partners

Five studies investigated whether index patients faced barriers to notifying partners [14,24,26,34,40]. Major perceived barriers in Peru included embarrassment, fear of rejection, stigma associated to the disease and difficulty in locating casual partners [26]. The main barriers for women to notify partners included: (1) their husband worked in a different town; (2) fear that their husband will accuse them of being the source of the STI; and (3) fear of divorce [14,26]. Married men reported barriers to notifying their wives being: (1) the wife lived in a rural area; (2) embarrassment; (3) fear of loss of respect; and (4) fear of disharmony in the family. Women mostly feared that their extramarital relationships would be revealed and/or they feared that they would be treated as the source of their husband's infection [40]. For men, the main barriers were fears of being considered unfaithful, leading to separation or divorce. Two major barriers for notifying casual and commercial partners included (1) being partners who were not traceable and (2) men did not care to notify them since they had no further plan to have a sexual relationship with the casual partner in question [14]. Women who had non-spousal partners indicated that they depended upon them financially and could not risk annoying them for fear of losing material support.

### Staff or investigator reported infrastructure barriers in notifying partners

Most common structural barriers reported in the studies included inadequate staff and lack of accurate, affordable diagnostic services. Inadequately trained staff in STD clinics was one of the major barriers making provider-oriented referrals difficult; increasing the work load of already overburdened health care providers was deemed unrealistic [16]. The socioeconomic profile of the study participants was considered a barrier to PN in some settings in that the population was often mobile, and lacked access to mail, telephones, or traceable addresses [16]. Lack of valid, user-friendly, and affordable STI diagnostic methods was also mentioned as structural barriers for PN, while syndromic management approaches mostly used in developing countries were reported to identify false positive cases leading to medicate people who do not need it[27,37]. Inadequate resources and poor availability of trained staff were reported as barriers of PN, especially provider-oriented notification in already overburdened health care systems [38]. Lacking of necessary counselor in the STD clinics was specifically mentioned as barrier to PN programs implementation [19,31].

### PN approaches that were evaluated in developing countries

Five intervention studies evaluated PN outcomes in developing countries [10,14,18,22,27]. Two randomized trials investigated effectiveness of client-centered counseling on PN outcomes [14,18]. The trial in Zimbabwe found that index patients in the counseling arm were more likely to report notifying their partners compared to the controls [92% vs. 67%, adjusted odds ratio (AOR) 4.1; 95% confidence interval (CI) 1.3-13.2; p < 0.001] adjusted for age, gender, employment status, and type of partners[14]. The trial in Zambia reported that an average of 1.8 partners per infected man were treated in the counseling-for-notification group compared to 1.2 in the control group (p < 0.001), but they did not find any significant effect of PN counseling among infected women [18]. One randomized trial investigated if patient-delivered medication was effective in treating partners of STI patients compared to patient-based referral alone. Medicines were reported as delivered to 74% of the partners in the patient-delivered medication group compared to verified successful referral to the clinics in just 34% of the patient-based partner referral group [risk ratio (RR) 2.44; 95% CI 1.95-3.07; p < 0.001] [22]. When given a choice, 85% chose patient-delivered partner medication while only 13% chose patient- based referral approach in one South African study [27]. Another South African study investigated if video-based health education would be effective in improving partner referral; the rate of contact cards returned per index patient was 0.27 in the intervention phase, compared with 0.20 in the control phase an increase that may have been due to chance [10].

## Discussion

Patient-oriented PN approaches were preferred methods in the developing countries as per our review studies, which is in accordance with the WHO recommendation that patient-based notification should be the first step for developing countries [6]. Counselling to index STI patients on PN was found to be effective in increasing partner referral in studies conducted in Zambia, Zimbabwe, and Kenya [14,16,18]. This simple intervention is reasonable for developing countries for several reasons: (1) overworked health care providers dealing with a large number of STI patients will not be suitable for provider-oriented notifications; (2) counselling services were well received by clients; (3) counselling for patient-oriented notifications can be easily integrated into public and private sector clinics; and (4) counselling costs will be lower than physician counselling [14]. The major challenge however, to this intervention is that counsellors are not available in health centres in most developing countries. A health system audit in rural South Africa, one of sub-Saharan Africa's middle income nations, reported that almost half of their study clinics did not have counsellors in their STD clinics [31]. Similarly, in China most STD clinics do not provide counselling services to their patients [19].

Randomized trial investigated patient-delivered partner medications approach in developing countries suggested that this approach was more effective in reaching sexual partners of index STI patients for treatment compared to patient-oriented notification alone [22]. Some other PN studies conducted in China, Kenya, South Africa, and Peru recommended further evaluation of the effectiveness of partner-delivered treatment approaches in developing countries because of low number of partners known to receive treatment for STIs after notification justify potential importance of expedited partner-facilitated therapy programs [16,27,26,45]. However, implementation of patient-delivered treatment approaches may conflict with medical practice guidelines in some countries, since antibiotics are given to patients to give to their sexual contacts without evaluation by a clinician. It is uncertain whether the treatments will reach the intended target patient, nor whether that person actually needs the therapy. A decision analysis model in a developing country context would be helpful to quantify the relative costs and benefits; a model crafted for high-cost, comparatively low prevalence circumstances suggested PN to be cost-effective even in the United States [46].

Major barriers to successful PN from the patient's perspective were grounded in cultural and psychosocial issues. The stigma associated with STIs discourages patients from discussing their infections and sexual behaviours with their partners, especially given extramarital sexual relationships [26,47]. Gender, power structure, and partner type were important in PN intentions and practices [24]. Fear of abuse and rejection resulting from partner referral was found to be major barriers, especially for women [14,26]. Notification of non-spouse partners by index patients was low both for men and women. Women who had non-spouse partners indicated that these partners were often concurrent and that they depended upon them financially. Economic vulnerability of women must be considered in the design of PN in developing countries, given women's difficulties in negotiating safer sex and broaching sexual matters [48].

STI diagnoses are predominantly based on syndromic approaches in most developing countries leaving possibilities of over diagnosis, and overmedication [49,50]. PN in such cases could result in unnecessary social harm to the patients, resulting partner violence, separation, and social isolation [27]. Weak health systems with under-capacitated STD clinics and limited personnel (including doctors, nurses and counsellors) are unlikely to emphasize PN when funds for medicines and other supplies are insufficient [16]. Exclusive facilities for STI/HIV management in developing countries are rare; STI clinical services are often merged with skin, other reproductive health and family planning services. Inadequate attention may be devoted to the STI patients when health care providers are overloaded with many other patients. Clinicians are inadequately trained in STI management and may be unmotivated to support PN efforts [51].

A formal meta-analysis was not feasible for this topic. There were substantial variations in study designs and in the populations they studied. Many approaches were used in the ascertainment of PN outcomes. Most of the studies were descriptive in nature; data were collected from STI patients or from STI service providers [30,32,52] from facility audits [53,54] and/or from qualitative observation of service delivery processes [35,36,53], and none of the review studies was found to be methodologically stronger according to our quality ratings. Ascertainment of PN outcomes came from self-reported data from index patients and from validation of clinic records to verify partner referral or partner treatment. While a meta-analysis would be meaningless in face of such disparate studies, it is nonetheless revealing that key themes were consistent across continents, populations, and study designs, suggesting their robustness. Some of the data presented in this study was absolute number, in systematic reviews such vote counting is known to frequently bias the interpretation of findings, since this ignores the effect size and sample size of the studies. However, such counting is mainly used to categorize the study characteristics not to report any study outcomes as such.

## Conclusions

Our review findings indicate that PN for STIs is feasible in developing countries and that most patients diagnosed with STIs are willing to self-notify their regular partners. Among the PN intervention strategies; counselling of index STI patients and patient delivered partner medication were found to be somewhat effective in Africa. Counselling of index STI patients is particularly useful in terms of raising awareness of PN, eliminating stigma and fear related to STIs, and should be promoted in both public and private STD clinics. Other innovative strategies should be explored and evaluated to increase PN and referral in developing countries. One example of such innovations is the use of cell phones in notifying partners, which is now widely accessible among urban and rural young adults in most developing countries [55]. Use of cell phones could be a tool to reach partners of index STI patients to inform them about possible exposure yet maintaining a good deal of confidentiality [56,57]. The internet may be a future venue for PN as access is growing in many developing countries [58,59]. STIs are not perceived as a major public health problem by the policy makers in most developing countries, resulting in inadequate resource allocation [10,60-66]. Well designed PN intervention to evaluate the impact on STI prevalence and incidence outcomes with cost effectiveness data may motivate policy makers in developing countries to pay more attention formulating policies to improve quality of care in STI service facilities to promote PN.

## Competing interests

The authors declare that they have no competing interests.

## Authors' contributions

NA collected, analyzed and interpreted his data with input from SK and EC. NA, KS, EC, SHV and SK have all: (1) contributed to the design of this study, (2) participated in the writing process of this article and (3) given their accord for the publication of this article.

## Pre-publication history

The pre-publication history for this paper can be accessed here:

http://www.biomedcentral.com/1471-2458/10/19/prepub
